# Metagenomic analysis of nepoviruses: diversity, evolution and identification of a genome region in members of subgroup A that appears to be important for host range

**DOI:** 10.1007/s00705-021-05111-0

**Published:** 2021-08-09

**Authors:** J. M. Hily, N. Poulicard, J. Kubina, J. S. Reynard, A. S. Spilmont, M. Fuchs, O. Lemaire, E. Vigne

**Affiliations:** 1 IFV, Le Grau-Du-Roi, France; 2 Université de Strasbourg, INRAE, SVQV, UMR-A 1131, F-68000 Colmar, France; 3 PHIM, Université Montpellier, IRD, INRAE, Cirad, SupAgro, Montpellier, France; 4 Institute for Plant Production Science, Agroscope, 1260 Nyon, Switzerland; 5 Cornell University, Geneva, NY USA

## Abstract

Data mining and metagenomic analysis of 277 open reading frame sequences of bipartite RNA viruses of the genus *Nepovirus*, family *Secoviridae*, were performed, documenting how challenging it can be to unequivocally assign a virus to a particular species, especially those in subgroups A and C, based on some of the currently adopted taxonomic demarcation criteria. This work suggests a possible need for their amendment to accommodate pangenome information. In addition, we revealed a host-dependent structure of arabis mosaic virus (ArMV) populations at a cladistic level and confirmed a phylogeographic structure of grapevine fanleaf virus (GFLV) populations. We also identified new putative recombination events in members of subgroups A, B and C. The evolutionary specificity of some capsid regions of ArMV and GFLV that were described previously and biologically validated as determinants of nematode transmission was circumscribed *in silico*. Furthermore, a C-terminal segment of the RNA-dependent RNA polymerase of members of subgroup A was predicted to be a putative host range determinant based on statistically supported higher π (substitutions per site) values for GFLV and ArMV isolates infecting *Vitis* spp. compared with non-*Vitis*-infecting ArMV isolates. This study illustrates how sequence information obtained via high-throughput sequencing can increase our understanding of mechanisms that modulate virus diversity and evolution and create new opportunities for advancing studies on the biology of economically important plant viruses.

**Supplementary Information:**

The online version contains supplementary material available at 10.1007/s00705-021-05111-0.

## Introduction

New viral sequences are being discovered at an unprecedented rate since the advent of high-throughput sequencing (HTS). The recovery of numerous complete or almost complete viral genome sequences from different ecosystems (*i.e.*, environmental, human, veterinary, plant) has allowed previously unknown virus genomes to be described and their diversity to be studied. This wealth of information is creating the possibility of using the pangenome for virus taxonomy [17] and increasing our understanding of the mechanisms that modulate virus diversity, evolution, vector and host specificity, and epidemiology [37]. However, new challenges arise, for instance, with regard to virus classification. Taxonomy traditionally relies not only on the genetic relationships among sequences of a few viral coding regions, primarily the replicase and/or coat protein coding domains, but also on biological properties such as vector species and host range, among other features [48]. This type of biological information is critical for the taxonomic classification of currently known plant viruses, but it is generally lacking when only metagenomic data are available.

Nepoviruses are plant picorna-like viruses belonging to the subfamily *Comovirinae* in the family *Secoviridae* [46]*.* Their transmission occurs in a non-persistent and non-circulative manner via ectoparasitic nematodes of the genera *Xiphinema*, *Longidorus*, and *Paralongidorus* [44]. Long-distance dissemination of nepoviruses occurs with the exchange of uncontrolled propagation material and the use of infected cuttings and budwood for grafting. Seed and pollen transmission have been documented for some, but not all, nepoviruses, and transmission by mites has been observed in rare cases. The genus *Nepovirus* includes 40 species whose members are widely distributed in temperate regions (https://talk.ictvonline.org/ictv-reports/ictv_online_report/positive-sense-rna-viruses/picornavirales/w/secoviridae/591/genus-nepovirus) [22]. Most nepoviruses have a broad natural host range, including annual herbaceous species (e.g., *Beta vulgaris*, *Nicotiana tabacum*, and *Solanum lycopersicum*) and perennial woody species (e.g., *Vitis vinifera*, *Prunus domestica*, *Rubus idaeus*, and *Olea europaea*), and cause significant crop losses worldwide [11].

The genome of nepoviruses is composed of two single-stranded, positive-sense RNAs (RNA1 and RNA2). Both genomic RNAs are necessary for infection *in planta*. These RNAs encode a large polyprotein, P1 for RNA1 and P2 for RNA2, which is cleaved by the viral proteinase into functional proteins [11]. P1 is the precursor of proteins that are necessary for replication, including a helicase with a nucleoside-triphosphate-binding domain, a proteinase (Pro), and an RNA-dependent RNA polymerase (Pol). Depending on the viral species, one (1A) or two (X1 and X2) proteins are located upstream of the helicase domain. The function of these proteins is not fully elucidated yet. P2 includes the coat protein (CP), multiple units of which form icosahedral virions with a diameter of 26-30 nm. The cell-to-cell movement protein (MP) domain is located immediately upstream of the CP domain. Depending on the nepovirus species, one (2A, which is required for the replication of RNA2) or two (X3 and X4 of unknown function) proteins are located upstream of the MP [13]. Three subgroups of nepoviruses have been recognized based on RNA2 properties, including its organization and size, phylogenetic relationships in the CP coding region, and cleavage sites recognized by the viral proteinase [11]. The three nepovirus subgroups are named A, B, and C.

One of the most important viral diseases of grapevines is infectious degeneration. This disease is caused by members of 15 different *Nepovirus* species [6, 43]. Most grapevine-infecting nepoviruses are generally restricted to a particular region of the world. For example, arabis mosaic virus (ArMV) is limited to European vineyards, while tobacco ringspot virus (TRSV), tomato ringspot virus (ToRSV), peach rosette mosaic virus (PRMV), and blueberry leaf mottle virus are present in American vineyards. In contrast, grapevine fanleaf virus (GFLV) is present in most vineyards worldwide.

The genetic diversity of nepoviruses has been analyzed extensively, primarily using information collected from RT-PCR-based studies combined with Sanger sequencing, generally in the CP coding region [14, 52]. Similarly, diversity studies and phylogenetic analysis have been reported for members of the family *Secoviridae*, including nepoviruses [45, 51]. However, several new nepoviruses have been characterized recently, and the number of complete genome sequences of nepovirus isolates has increased exponentially in the past five years [1, 2, 4, 12, 15, 18, 23–25, 41, 50, 55, 56, 58]. In this study, we built on these latest advancements in nepovirus research and carried out metagenomic analysis. We focused on RNA1 and RNA2 coding sequences to gain new insights into viral diversity and evolution, and we identified a hitherto undescribed conserved region of the genome that is putatively involved in determining the host range of two subgroup A nepoviruses.

## Materials and methods

### Sequence analysis, genetic diversity, and detection of recombination

The complete nepovirus ORF1 and ORF2 sequences were retrieved from NCBI as of January 2020, our own curated nepovirus sequence repository obtained by analysis of high-throughput or Sanger sequencing datasets, and a selection of Sequence Research Archive datasets from GenBank [19]. In total, 110 ORF1 sequences and 167 ORF2 sequences were used in this study (Supplementary Tables S2 and S3). In addition, sequences of specific domains were retrieved from NCBI (see Supplementary Table S8).

Codon-based multiple sequence alignments and maximum-likelihood (ML)-based phylogenetic trees were prepared using MUSCLE [7], implemented in MEGA7 and MEGAX software [26, 27], excluding the viral untranslated regions (UTRs). The best ML-fitted model for each sequence alignment was used, and nodes in phylogenetic trees were validated by bootstrap analysis (100 replicates). For visualization effects, FigTree v. 1.3.1 was used (http://tree.bio.ed.ac.uk/). The diversity index (π), which is the average number of nucleotide substitutions per site between any two sequences in a multi-sequence alignment, and the variation of π along genome sequences was evaluated by sliding window analysis (length, 80; step size, 20) using DnaSP v.6.12.03 [29] and MEGA X.

A search for potential recombination signals was performed using all seven algorithms implemented in RDP v4.97 (RDP4) [32]. The default settings were used for each algorithm, and only recombination events detected by five or more methods were considered.

Differences in nucleotide sequence diversity of viral populations defined using different modalities were tested by analysis of molecular variance (AMOVA), as implemented in Arlequin v. 5.3.1.2 [10]. AMOVA calculates the Fixation index, F_ST_ index explaining the between-groups fraction of total genetic diversity. The significance of these differences was evaluated by performing 1000 sequence permutations.

Tajima’s D (*D*_*T*_) and sliding window analyses were conducted using DnaSP v. 6.12.03 [29] in order to distinguish the viral populations evolving randomly (per mutation-drift equilibrium; *D*_*T*_ = 0) from those evolving under a nonrandom process (*D*_*T*_ > 0: balancing selection, sudden population contraction; *D*_*T*_ < 0: recent selective sweep, population expansion after a recent bottleneck).

## Results and discussion

### Phylogenetic relationships among nepoviruses

Only complete open reading frame (ORF) sequences of RNA1 (ORF1) and RNA2 (ORF2) of nepoviruses were considered in this study. All sequences were retrieved from the NCBI database as of January 2020, our own curated nepovirus sequence repository, and a selection of Sequence Read Archive (SRA) datasets from the GenBank database. Data mining was performed to increase the number of sequences for ArMV, GFLV, and mulberry mosaic leafroll-associated virus (MMLRaV), a novel nepovirus [31], as described previously [19]. These data mining, Sanger, or Illumina sequencing efforts resulted in 46 new sequences (24 for RNA1 and 22 for RNA2) of ArMV, GFLV, and MMLRaV. New sequences were deposited in the GenBank database (Supplementary Table S1). In total, both genomic RNA sequences were recovered from members of 29 nepovirus species, except from olive latent ringspot virus, for which only a single RNA2 sequence but no RNA1 sequence is available (Table 1). Two nepoviruses (GFLV and ArMV) made up the majority of sequences analyzed in this study, while most species were represented by one or a few sequences of either genomic RNAs (Table 1). Novel nepoviruses used in this study included MMLRaV [31], caraway yellows virus [12], potato virus B [4], and red clover nepovirus A [25]. A few new viruses and isolates belonging to the genus *Nepovirus* have been identified since we last consulted NCBI (January 2020). The corresponding sequences were not included in this study (Supplementary Table S10). In addition, a few viruses described in the literature as potential members of new nepoviral species, such as Hobart nepovirus 3 [42] or Zhuye pepper nepovirus 1 [3], were not taken into account in this study because the sequences were incomplete or discrepancies were observed between the datasets available at NCBI and the publications. Furthermore, only a single sequence was chosen from a group of sequences displaying nucleotide sequence identity higher than 99% unless the isolates were from different hosts and/or different countries. Complete lists of the 110 ORF1 and 167 ORF2 sequences selected for this study are provided in Supplementary Tables S2 and S3, respectively.

**Table 1:** List of nepoviruses used in this study

Virus name	Abbreviation	Subgroup	ORF1 (N seq.)	ORF2 (N seq.)	ORF1 (length)	ORF2 (length)	References
Aeonium ringspot virus	AeRSV	a	1	1	2314 aa	1128 aa	[54]
Arabis mosaic virus	ArMV	A	17	21	2282-2285 aa	1041–1122 aa	[27]
Arracacha virus A	AVA	A	1	1	2376 aa	1137 aa	[27]
Grapevine deformation virus	GDefV	A	1	1	2284 aa	1107 aa	[27]
Grapevine fanleaf virus	GFLV	A	40	80	2284 aa	1107–1118 aa	[27]
Mulberry mosaic leaf roll associated virus	MMLRaV	a	3	3	2103 aa	1092–1093 aa	[36]
Melon mild mottle virus	MMMoV	a	1	1	2314 aa	1120 aa	[50]
Olive latent ringspot virus	OLRSV	A	0	1	na	1145 aa	[27]
Petunia chlorotic mottle virus	PCMoV	a	1	1	2316 aa	1119 aa	[4]
Potato black ringspot virus	PBRSV	A	1	4	2324 aa	1078-1082 aa	[27]
Tobacco ringspot virus	TRSV	A	2	3	2303-2304 aa	1101 aa	[27]
Raspberry ringspot virus	RpRSV	A	2	4	2366-2367 aa	1106–1107 aa	[27]
Artichoke Italian latent virus	AILV	B	1	3	2280 aa	1347 aa	[27]
Beet ringspot virus	BRSV	B	3	4	2266-2271 aa	1350–1357 aa	[27]
Cycas necrotic stunt virus	CNSV	B	4	5	2283-2338 aa	1240*–1357 aa	[27]
Grapevine Anatolian ringspot virus	GARSV	B	1	1	2243 aa	1350 aa	[27]
Grapevine chrome mosaic virus	GCMV	B	1	3	2250 aa	1324–1325 aa	[27]
Potato virus B	PVB	b	1	1	2264 aa	1371 aa	[7]
Red clover nepovirus A	RCNVA	b	2	3	2257 aa	1135*–1366 aa	[30]
Tomato black ring virus	TBRV	B	3	4	2266-2268 aa	1343–1344 aa	[27]
Blackcurrant reversion virus	BRV	C	1	1	2094 aa	1626 aa	[27]
Blueberry latent spherical virus	BLSV	c	1	1	2172 aa	1631 aa	[28]
Caraway yellows virus	CawYV	c	1	1	2213 aa	1704 aa	[15]
Cherry leaf roll virus	CLRV	C	11	10	2109-2113 aa	1589–1641 aa	[27]
Grapevine Bulgarian latent virus	GBLV	C	1	1	2095 aa	1499 aa	[27]
Peach rosette mosaic virus	PRMV	C	2	1	2150-2167 aa	1474 aa	[27]
Potato virus U	PVU	C	1	1	1935 aa	1544 aa	[27]
Tomato ringspot virus	ToRSV	C	5	5	2191-2200 aa	1882–1979 aa	[27]
Soybean latent spherical virus	SLSV	c	1	1	2195 aa	1398 aa	[62]
	Total		110	167			

Members of each subgroup (A, B, and C) are indicated by a capital letter if the species to which the virus belongs has been officially ratified by ICTV, or in lowercase when ICTV ratification is pending. The number of ORF1 and ORF2 sequences used for each species is shown. The length of both ORFs is indicated for each virus. * indicates a shorter sequence for an isolate of cycas necrotic stunt virus (reference isolate) and red clover nepovirus A. na, not applicable

**Fig. 1:**
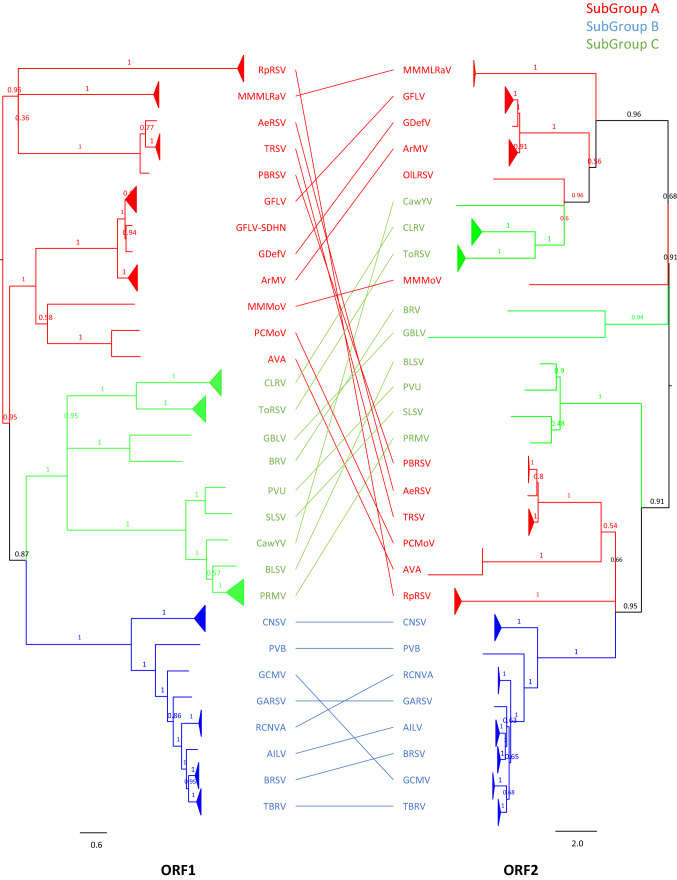
Tanglegram of maximum-likelihood phylogenetic trees inferred from 110 ORF1 and 167 ORF2 nucleotide sequences of nepoviruses. Colors represent the three nepovirus subgroups: red for subgroup A sequences, blue for subgroup B sequences, and green for subgroup C sequences. Clades with several sequences from the same species are collapsed. Numbers at nodes indicate bootstrap values based on 100 replicates. The scale bar corresponds to the number of substitutions per site.

Nucleotide sequence comparisons confirmed the classification of nepoviruses into three subgroups with higher inter-subgroup than intra-subgroup mean distance values (Table 2). Subgroup B sequences displayed the lowest maximum pairwise distance values, which were well below the inter-subgroup mean distance values, suggesting a well-defined group of virus isolates (Table 2). The inter-subgroup mean distance value was lower than the maximum intra-subgroup mean distance value for subgroup A and C sequences, revealing a greater variability and less well-defined groups of virus isolates (Table 2). After performing an alignment of ORF1 and ORF2 sequences, phylogenetic trees were constructed by the maximum-likelihood (ML) method, using the best-fit model (GTR+G+I) (Fig. 1). Interestingly, the members of each subgroup were separated better in the tree based on ORF1 than the one based on ORF2 sequences (Fig. 1 and Supplementary Fig. S1). Indeed, the ORF1 nucleotide sequences of virus isolates of subgroups A, B, and C clustered in separate and well-supported clades in a tanglegram (Fig. 1) and in an unrooted cladogram (Supplementary Fig. S1). Nucleotide sequences of nepovirus isolates of subgroup B were also well defined when using ORF2 sequences, but subgroup A and C ORF2 sequences were scattered in different clades in a tanglegram (Fig. 1) or unrooted cladogram (Supplementary Fig. S1). These results suggest that the classification of nepoviruses into distinct subgroups is more robust when based on ORF1 sequences than when based on ORF2 sequences. This finding should be considered by the International Committee on Taxonomy of Viruses (ICTV) *Secoviridae* Study Group to eventually define new demarcation criteria for nepoviruses when using pangenome information.

**Table 2:** Genetic distance within and between subgroups (SubGP) A, B, and C of the genus *Nepovirus*

	Within subgroup	N	Mean		Max. pairwise distance		Between subgroup	SubGP_A	SubGP_B	SubGP_C
			distance	*SE*						
ORF1	Overall	110	**0.485**	*0.003*	0.645					
	SubGP_A	70	**0.321**	*0.003*	0.615	ORF1	SubGP_A	–	*0.004*	*0.004*
	SubGP_B	16	**0.386**	*0.003*	0.527		SubGP_B	**0.613**	–	*0.004*
	SubGP_C	24	**0.463**	*0.003*	0.615		SubGP_C	**0.615**	**0.614**	–
ORF2	Overall	167	**0.476**	*0.003*	0.719					
	SubGP_A	121	**0.317**	*0.003*	0.689	ORF2	SubGP_A	–	*0.005*	*0.004*
	SubGP_B	24	**0.416**	*0.003*	0.573		SubGP_B	**0.668**	–	*0.004*
	SubGP_C	22	**0.542**	*0.003*	0.697		SubGP_C	**0.643**	**0.688**	–

The mean nucleotide distance is shown in bold, and the standard error (SE) is shown in italics. The maximum value of pairwise distance within subgroups is shown. "N" represents the number of sequences used for calculation

### New challenges for species identification within the genus *Nepovirus*

**Fig. 2:**
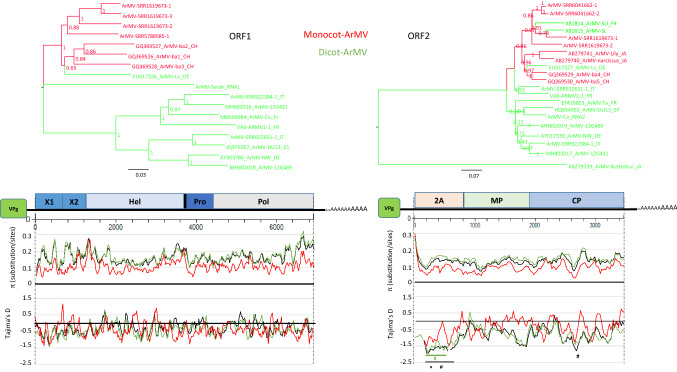
Phylogenetic and diversity analysis of arabis mosaic virus (ArMV) isolates from different plants, using a corpus of 17 ORF1 (left panel) and 21 ORF2 (right panel) nucleotide sequences. Colors correspond to hosts, with red for monocotyledonous plants, green for dicotyledonous plants, and black for all plants. Maximum-likelihood phylogenetic trees are shown. Numbers at each node indicate bootstrap values based on 100 replicates, and scale bars show genetic distance. Graphics represent π (substitutions) and Tajima’s D (*D*_*T*_) for evolution along the ORF1 and ORF2 sequences. Colored bars with # and * correspond to statistically validated regions (*P*-values at 0.05 and 0.001), respectively

Species demarcation criteria for nepoviruses have been defined by the ICTV (https://talk.ictvonline.org/ictv-reports/ictv_online_report/positive-sense-rna-viruses/picornavirales/w/secoviridae). These include CP amino acid (aa) sequence identity less than 75% and conserved protease-polymerase (Pro-Pol) region aa sequence identity less than 80%, among other criteria. We assessed whether these two major demarcation criteria are applicable to the corresponding complete ORF1 and ORF2 aa sequences. Some discrepancies with regard to the intra-species aa sequence identity falling outside the species demarcation were observed for PRMV ORF1 sequences (78.99%) and ORF2 sequences of cherry leaf roll virus (CLRV), ToRSV, cycas necrotic stunt virus, and ArMV (below 74.05%) (Supplementary Table S4). These results revealed that analyzing complete ORF sequences may be problematic for the establishment of new virus species and the classification of new genetic variants of members of existing virus species if the current demarcation criteria pertaining to partial genome sequence information were to be applied. The results further suggest that the demarcation criteria for species in the genus *Nepovirus* should be amended to accommodate pangenome information. In addition, the ORF2 sequence of ArMV isolate Butterbur was a clear outlier among the ArMV isolates with lower identity values at both the nucleotide (Fig. 2, Supplementary Figs. S3 and S5) and amino acid (70.25%, Supplementary Table S4) levels. According to the original report [21], the pathological and serological features of ArMV-Butterbur are unique, and its CP is 504 aa long (as for all GFLV CPs), while all other ArMV CPs are 505 aa long. These features underscore the need for additional work to ascertain the taxonomic position of ArMV-Butterbur and its recognition as an isolate of ArMV, in particular since no RNA1 sequence is available.

Similarly, the ORF1 aa sequence identity between some isolates belonging to different species was higher than 80%, for example, for beet ringspot virus (BRSV) and tomato black ring virus (TBRV), as well as for BRSV and artichoke Italian latent virus (AILV) (Supplementary Table S4). This high level of sequence similarity could also explain the large number of inter-species recombination events identified between members of these particular species (see the dedicated section below). However, inter-species diversity was below the species demarcation level (< 75%) for ORF2 sequences, unambiguously defining BRSV, TBRV, and AILV as members of different species (Supplementary Table S4). One particular case of interest is grapevine deformation virus (GDefV) [20], a subgroup A nepovirus. GDefV ORF2 aa sequences display 73 and 71% identity to those of GFLV and ArMV, respectively [16], and GDefV ORF1 aa sequences have higher identity to those of GFLV (86-89%) than to those of ArMV (73-74%) [8]. According to the species demarcation criteria for nepoviruses, GDefV would be classified as a highly divergent variant of GFLV when focusing on the ORF1 sequences but as a member of a new species based on ORF2 sequences.

### Identification of putative recombination events within and between nepovirus species

Putative intra-species recombination events have been extensively reported for nepoviruses, mostly in the GFLV RNA2-encoded MP and CP domains [34, 35, 38, 49, 52–54]. Recombination events have also been described for ToRSV [56] and grapevine chrome mosaic virus (GCMV) [5]. In addition, many inter-species recombination events have been detected, mostly between ArMV and GFLV [9, 34, 35, 54], but also between GCMV and TBRV [5, 28]. With the use of HTS and the recovery of complete virus genome sequences, recombination events can be detected all along the two genomic RNAs [18]. Here, we used the same corpus of nepovirus sequences and searched for potential recombination events using the RDP4 program. Recombination events were only considered when predicted by five or more algorithms with *P*-values < 10^-3^ (Table 3, Supplementary Tables S5 and S6).

**Table 3:** Number of putative intra- and inter-species recombination events predicted by RDP4 for members of the three nepovirus subgroups (SubGP A, B, and C)

	Intra-species	Inter-species
ORF1	51	16
SubGP_A	43	1
SubGP_B	0	12
SubGP_C	8	3
ORF2	93	144
SubGP_A	89	122
SubGP_B	2	19
SubGP_C	2	1

Detailed information on the genomic location of recombination events, major and minor parents, and *P*-values is provided in Supplementary Tables S5 and S6

Potential intra-species recombination events were identified in ORF1 and ORF2 sequences, mostly of subgroup A members (Table 3, Supplementary Table S5 and Supplementary Fig. S2). Almost twice as many putative recombination events were predicted in ORF2 sequences than in ORF1 sequences (Table 3). Both of these observations most definitely reflect the total number of sequences being recovered and used in this study. Many recombination events were predicted in GFLV and ArMV sequences, with some hotspots, *i.e*. more than one putative recombinant per site (Supplementary Table S5 and Supplementary Fig. S2). In addition, putative recombination events were also identified for the first time for AILV, CLRV, and raspberry ringspot virus.

All inter-species recombination events predicted in this study strictly involved members of the same subgroup (Supplementary Table S6). Surprisingly, the number of inter-species recombination events was higher than the number of intra-species recombination events within ORF2 sequences (Table 3). For example, 31 inter-species and two intra-species recombination events were detected for subgroup B ORF2 sequences (Table 3 and Supplementary Fig. S2). It should also be noted that all putative ORF1 recombination events detected for subgroup B involved members of different species (Supplementary Table S6). In contrast, all subgroup A recombination events that were predicted involved only ArMV, GFLV, and GDefV (Fig. 1 and Supplementary Fig. S1), again emphasizing their kinship. Recombination may have been facilitated for these three viruses because they have the potential to co-exist in grapevine, a common host, for long periods of time, thus increasing the likelihood of an potential encounter in the same host cell.

### Genetic diversity and population differentiation of ArMV from mono- and dicotyledonous plants

ArMV is a ‘generalist’ with a very broad natural host range, including winter barley, narcissus, *Ligustrum vulgare*, weeds, hops, berries, olive trees, apricot trees, and grapevines, among other species [14, 33, 40]. Our data mining efforts resulted in the retrieval of 17 complete ORF1 sequences from seven monocotyledonous plants and 10 dicotyledonous plants, as well as 21 complete ORF2 sequences from eight monocotyledonous plants and 13 dicotyledonous plants (Supplementary Tables S1, S2, S3, and S8).

The overall genetic diversity (π) of ArMV ORF1 and ORF2 was 0.166 ± 0.003 and 0.133 ± 0.003, respectively (Table 4). As observed previously [57], the coding region 2A is the most divergent genomic region, showing the highest diversity at the extreme 5’ end of ORF2 (Fig. 2), mostly due to size differences among isolates. For ArMV ORF1, the extreme 3’ end is the most divergent genomic region. A comparative analysis of ArMV sequences obtained from mono- and dicotyledonous plants revealed a significantly higher diversity in sequences from isolates infecting dicotyledonous plants compared to isolates infecting monocotyledonous plants (0.170 ± 0.003 vs. 0.109 ± 0.003 and 0.145 ± 0.003 vs. 0.093 ± 0.003, for ORF1 and ORF2 sequences respectively; Table 4). When looking at the evolution pattern (Tajima’s D) of ORF1 sequences (Fig. 2), values were negative but close to 0 (D_T_ =  – 0.344; *P* > 0.1), suggesting that the population of ArMV is evolving as per mutation-drift equilibrium with no specific region under selection. On the other hand, two distinct regions of ORF2 sequences were under selection with an overall D_T_ value of  – 0.994 (*P* > 0.1) (Fig. 2). One of these two regions covers an aa stretch between two proline-rich segments of the central coding region of the 2A domain. The other region is a specific segment of the CP coding region that overlaps the previously defined R4 region, which is involved in specific transmission of ArMV by the nematode vector *Xiphinema diversicaudatum* [47].

**Table 4:** Genetic diversity for both ORFs of arabis mosaic virus (ArMV) and grapevine fanleaf virus (GFLV) isolates

		N	π ± SE	*D*_*T*_ (*P*-value)
ArMV	ORF1-overall	17	0.166 ± 0.003	– 0.346 (> 0.1)
	ORF1-monocot	7	0.109 ± 0.003	– 0.438 (> 0.1)
	ORF1-dicot	10	0.170 ± 0.003	– 0.419 (> 0.1)
	ORF1-non-Vitis	9	0.109 ± 0.003	– 0.365 (>0.1)
	ORF1-Vitis	8	0.163 ± 0.003	– 0.346 (>0.1)
	ORF2-overall	21	0.133 ± 0.003	– 0.994 (> 0.1)
	ORF2-monocot	8	0.093 ± 0.003	– 0.441 (> 0.1)
	ORF2-dicot	13	0.145 ± 0.003	– 0.969 (> 0.1)
	ORF2-non-Vitis	10	0.137 ± 0.003	– 1.100 (>0.1)
	ORF2-Vitis	11	0.128 ± 0.003	– 0.616 (>0.1)
GFLV	ORF1-overall	40	0.127 ± 0.002	– 0.758 (>0.1)
	ORF1-FR	19	0.107 ± 0.002	– 0.387 (>0.1)
	ORF1-RoTW	21	0.139 ± 0.002	– 0.738 (>0.1)
	ORF1-Old	30	0.124 ± 0.002	– 0.735 (>0.1)
	ORF1-New	10	0.131 ± 0.002	– 0.553 (>0.1)
	ORF2-overall	80	0.130 ± 0.005	– 0.783 (>0.1)
	ORF2-FR	22	0.097 ± 0.004	– 0.331 (>0.1)
	ORF2-RoTW	58	0.137 ± 0.005	– 0.740 (>0.1)
	ORF2-Old	48	0.129 ± 0.004	– 0.590 (>0.1)
	ORF2-New	32	0.127 ± 0.004	– 0.836 (>0.1)
	ORF2-Eu	42	0.120 ± 0.004	– 0.641 (>0.1)
	ORF2-Am	26	0.118 ± 0.004	– 0.712 (>0.1)
	ORF2-As	7	0.136 ± 0.005	– 0.183 (>0.1)
	ORF2-IT	7	0.140 ± 0.005	– 0.475 (>0.1)
	ORF2-SL	6	0.074 ± 0.003	1.618 (>0.1)
	ORF2-US-CA	17	0.120 ± 0.004	– 0.345 (>0.1)
	ORF2-CH	5	0.156 ± 0.006	– 0.399 (>0.1)
	ORF2-CL	9	0.119 ± 0.004	– 0.709 (>0.1)
	ORF2-FE	5	0.111 ± 0.005	– 0.107 (>0.1)

Overall diversity index (π) ± standard error (SE) and Tajima’s *D* (D_T_) with associated *P*-values based on N (number of sequences per group) are shown. Sequence populations were grouped according to the plant type or the geographic origin of the isolates (monocot, monocotyledonous; dicot, dicotyledonous; *Vitis*; non-*Vitis*; Old, Old World (France, Hungary, Italy, Switzerland); New, New World (Canada, USA, China, South Africa); FR, France; RoTW, rest of the world other than FR; EU, Europe; Am, Americas; As, Asia; IT, Italy; SL, Slovenia; CA, Canada; CH, Switzerland; CL, Chile; FE, Far East. The geographic origin of ArMV and GFLV isolates is specified for each sequence in Supplementary Tables S2 and S3

In a previous study [57], ArMV isolates were separated by the size and aa sequence identity of protein 2A into four groups (I to IV). Here, we recovered 43 ArMV 2A nucleotide sequences from GenBank (Supplementary Table S8) and confirmed the existence of three major clades corresponding to groups II, III, and IV (Supplementary Fig. S3). Group I was composed of a single sequence located within the group II clade. The sequences belonging to each group were genetically different with a high fixation index (F_ST_ ≥ 0.530) and strong statistical support (*P* ≤ 10^-5^) (Table 5). However, the size of the 2A domain was not linked to the plant host, with ArMV isolates from grapevine belonging to all four groups. A comparative analysis of 2A coding sequences from mono- and dicotyledonous plants documented a statistically supported genetic differentiation (F_ST_) (Table 5). Genetic differentiation according to mono- and dicotyledonous plants was also observed when looking at other RNA1 or RNA2 coding region sequences or the complete ORF1 and ORF2 sequences (Table 5, Supplementary Tables S7, S8 and Supplementary Fig. S3). Distinct F_ST_ values between mono- and dicotyledonous plants were also found at lower cladistic levels, strongly suggesting a likely genetic bias based on the plant host (Supplementary Table S7). Interestingly, similar results have been reported for CLRV, another generalist virus within the genus *Nepovirus* for which a host-species-dependent population structure was documented using only a short 375-bp sequence corresponding to the extreme 3’ part of the 3’ untranslated region [39].

**Table 5:** Genetic differentiation of arabis mosaic virus (ArMV) populations for the complete ORFs and the different coding regions

	Pop. comparisons	Fst	*P*-value	N
ORF1	Monocots vs. dicots	0.295	0.001	17
1A	Monocots vs. dicots	0.261	0.001	17
Hel	Monocots vs. dicots	0.132	0.006	17
VPg	Monocots vs. dicots	0.418	0.001	17
Pro	Monocots vs. dicots	0.253	0.001	17
Pol	Monocots vs. dicots	0.214	< 0.000	17
ORF2	Monocots vs. dicots	0.128	0.001	25
CP	Monocots vs. dicots	0.095	0.001	50
MP	Monocots vs. dicots	0.084	< 0.000	29
2A	Monocots vs. dicots	0.100	0.001	43
2A	II vs. III	0.587	< 0.000	31
2A	II vs. IV	0.551	< 0.000	23
2A	III vs. IV	0.530	< 0.000	28

The fixation index (F_ST_) with its associated *P*-value (*P*-values are significant when < 0.05) and the number of sequences (N) are indicated. Sequences were grouped by either the plant type (monocotyledonous versus dicotyledonous) or the size of the 2A coding region (groups II, III and IV)

### Genetic diversity and population differentiation of GFLV from different geographic regions

**Fig. 3:**
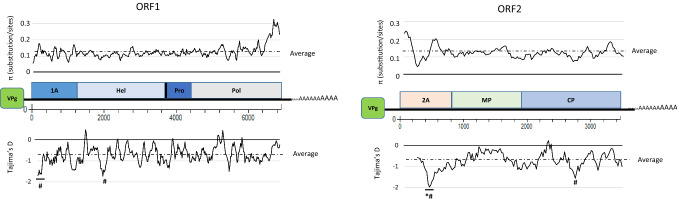
Genetic diversity analysis of grapevine fanleaf virus (GFLV) isolates, using a corpus of 40 ORF1 (left panel) and 80 ORF2 (right panel) nucleotide sequences. Graphics represent π (substitutions) and Tajima’s D (*D*_*T*_) for evolution along the ORF1 and ORF2 sequences. Bars and # and * correspond to statistically validated regions (*P*-values at 0.05 and 0.001), respectively

GFLV primarily infects *Vitis* spp., making the virus very specialized to this woody plant. The overall nucleotide sequence diversity for GFLV ORF1 (π = 0.127 ± 0.002) and ORF2 (π = 0.130 ± 0.005) sequences was very similar (Table 4). Plotting π along ORF1 sequences (Fig. 3) showed a highly divergent region at the 3’ end of the Pol domain. This result is consistent with other analyses of this particular aa stretch of P1, which was predicted to form an α-helix [18, 36]. Another highly polymorphic region was detected at the extreme 5’ end of ORF2 (Fig. 3), corresponding to a region where intra- and inter-species recombination events have been predicted (see above section and [54]). On the other hand, similar to ArMV, a significant drop in nucleotide sequence diversity is observed within a segment of 2A sequences located between two highly conserved proline-rich regions. The evolution of this particular domain of ORF2 sequences is not neutral, with statistical D_T_ values well below 0 (Fig. 3), indicating conservative selection with regard to the remainder of the ORF2 sequence. Similarly to ArMV, the same trend was observed for the R4 region of the CP domain [47]. Interestingly, these two regions, which display the lowest D_T_ values, suggesting a recent selective sweep, were mostly located in sequences recovered from grapevines from the New World (Supplementary Fig. S4). Regarding the evolution pattern of GFLV ORF1 sequences, values were negative but very close to 0 (D_T_ =  – 0.758), with two sites under selection (*P* > 0.1). The first site is located at the extreme 5’ end and the second site is positioned within the Hel domain (Fig. 3). When looking at the evolutionary pattern of P1 and P2 (dN-dS), most of the codons were under negative or neutral pressure (data not shown), as described previously [51].

No major differences were observed when separating GFLV sequences by geographic region (France vs. the rest of the world or Old vs. New World), with very similar π and D_T_ values (Table 4 and Supplementary Fig. S4). However, a genetic structuration between geographic regions was observed, although the F_ST_ values were extremely low (Supplementary Table S9), with differences in the evolution pattern of GFLV isolates from different parts of the world. This was even more noticeable when separating ORF2 sequences by continent (*i.e.*, Europe, Americas [combining North and South America] and Asia [Far East, Turkey, and Russia]). All F_ST_ values were statistically supported (*P* < 0.001). However, the disparity in F_ST_ values indicated that European and American GFLV variants were more closely related to each other than to the Asian variants. This observation was confirmed when grouping sequences into seven countries or specific regions of the world (France, Slovenia, Italy, USA, Chile, Far East and Switzerland). Some F_ST_ values were very high, underlying a strong genetic structuration among regions of the world, as confirmed when comparing sequences from the Far East (Iran and China) with other regions of the world (Table S9). This genetic differentiation according to Far East GFLV populations was previously described using GFLV MP sequences [49]. Altogether, these observations suggest a specific geographic evolution and genetic structuration of the virus.

### Common characteristics and major differences between grapevine-infecting ArMV and GFLV isolates

**Fig. 4:**
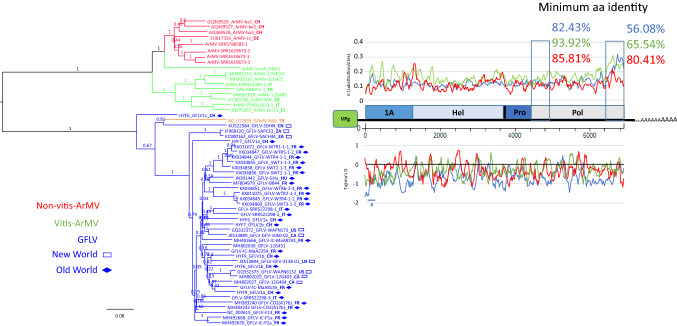
Phylogenetic and diversity analysis of grapevine fanleaf virus (GFLV), arabis mosaic virus (ArMV), and grapevine deformation virus (GDefV), using a corpus of 58 ORF1 nucleotide sequences. GFLV isolates are shown in blue, GDefV isolates in peach, ArMV isolates from grapevines (*Vitis*-ArMV) in green, and ArMV isolates from other plants (non-*Vitis* ArMV) in red. The country of origin of the isolate, if known, is indicated in bold at the end of the sequence name by two letters corresponding to the international ISO country code. GFLV sequences from the Old World (Turkey, Iran, France, Hungary, Germany, Slovenia, Russia, Italy, and Switzerland) are indicated by a solid diamond, and those from the New World are indicated by an open rectangle (Canada, USA, Chile, China, and South Africa). Maximum-likelihood phylogenetic trees are shown. Numbers at each node indicate bootstrap values based on 100 replicates, and the scale bar shows genetic distance. Graphics represent π (substitutions per site) and Tajima’s D (D_T_) for evolution along the ORF1 sequence. Percentages correspond to minimum aa identity for both framed regions within the polymerase domain. The colored bar with # corresponds to a statistically validated region at 0.05

**Fig. 5:**
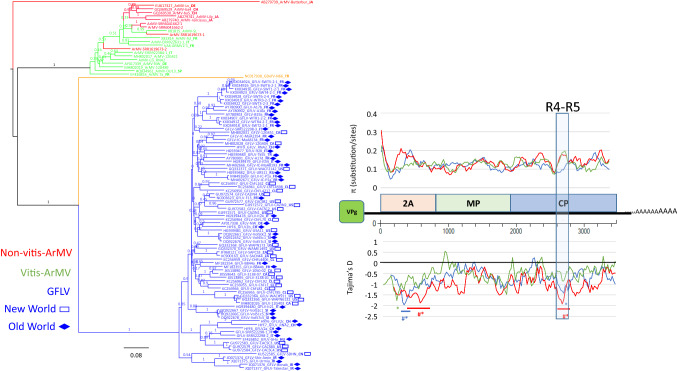
Phylogenetic and genetic diversity analysis of grapevine fanleaf virus (GFLV), arabis mosaic virus (ArMV), and grapevine deformation virus (GDefV), using a corpus of 102 ORF2 nucleotide sequences. GFLV isolates are shown in blue, GDefV in peach, ArMV isolates from grapevines (*Vitis*-ArMV) in green, and ArMV isolates from other plants (non-*Vitis* ArMV) in red. The country of origin of the isolate, if known, is indicated in bold at the end of the sequence name by two letters corresponding to the international ISO country code. GFLV sequences from the Old World (Turkey, Iran, France, Hungary, Germany, Slovenia, Russia, Italy, and Switzerland) are indicated by a solid diamond, and those from the New World are indicated by an open rectangle (Canada, USA, Chile, China, and South Africa). Maximum-likelihood phylogenetic trees are shown. Numbers at each node indicate bootstrap values based on 100 replicates, and the scale bar shows genetic distance. Graphics represent π (substitutions per site) and Tajima’s D (D_T_) for evolution along the ORF2 sequence. The boxed area corresponds to the R4-R5 region of the CP domain. Colored bars with # and * correspond to statistically validated regions (*P*-values at 0.05 and 0.001), respectively

ArMV and GFLV are very closely related but belong to different species (Fig. 1). They share many characteristics such as hosts (*i.e.*, grapevine), closely related vectors (*Xiphinema* spp.), similar symptomatology, and many natural inter-species recombinants. While genetically different (Supplementary Table S4, Figs 4 and 5), similar patterns in their respective genetic diversity were observed along ORF1 sequences, especially when separately analyzing sequences from *Vitis*-infecting ArMV isolates from non-*Vitis*-infecting ArMV isolates. As detailed above, one of the hallmarks of GFLV is a higher π value at the C-terminus of Pol. A higher π at the C-terminal end of Pol was also clearly identified in *Vitis*-infecting isolates, but not in non-*Vitis*-infecting ArMV isolates (Fig. 4). Such specific increased genetic diversity in *Vitis*-infecting ArMV and GFLV isolates was not due to the overlap of a hidden ORF (Supplementary Fig. S6), as described for sobemoviruses [30]. This diversity was also observed at the aa level, with a percent identity higher than 80.41% in the case of non-*Vitis*-infecting ArMV isolates, but as low as 65.54% and 56.08% for *Vitis*-infecting ArMV and GFLV isolates, respectively (Fig. 4). Such high divergence was not found when specifically looking at the first 148 aa of the Pol domain, where the sequence identity was above 82%. While highly divergent between ArMV and GFLV (Supplementary Fig. S7), the Pol C-terminus has only two amino acids that are mostly conserved between *Vitis*-infecting ArMV and GFLV isolates, at position 683 and 746 (Supplementary Fig. S8). Could these two residues be implicated in host adaptation mechanisms? More work is needed to address this hypothesis.

The ORF2 sequences of ArMV and GFLV have many characteristics in common (Fig. 5). For example, higher genetic diversity is detected at the 5’ end of 2A, partly as a result of indels. However, major differences between these viruses were found when focusing specifically on the CP domain, with a clearly different level of genetic diversity in the R4-R5 region between ArMV and GFLV (Fig. 5 and Supplementary Fig. S5). This region is important for vector transmission [47]. When looking at the evolution pattern, most of the ORF2 sequences seem to be evolving randomly, while two regions display a non-random evolution pattern. One of these two regions corresponds to the 2A domain, and the other to the R4-R5 region within the CP domain, both showing strong constraints.

## Conclusion

Data mining and metagenomic analysis of complete ORF sequences has provided new insights into the diversity of viruses in the genus *Nepovirus*, family *Secoviridae*, with a special emphasis on GFLV and ArMV, the two most important viruses involved in degeneration disease of grapevine in France. Our results confirmed a probable phylogeographic structure of GFLV populations and revealed a host-dependent structure of ArMV populations at a cladistic level. The C-terminus of the RNA-dependent RNA polymerase of GFLV and ArMV is predicted to be a potential host range determinant. More work is needed to test this hypothesis biologically. Furthermore, some of the current species demarcation criteria that are applied to limited genomic regions may not be validated for all nepoviruses at the ORF sequence level. This suggests the need to adapt some of the taxonomic criteria to pangenome information. Nonetheless, with an ever-increasing amount of sequence data obtained through HTS, there are new opportunities for studying nepovirus biology, characterizing nepoviral communities in plants, improving nepovirus taxonomy, and exploiting pangenomic and populational information for developing anti-viral strategies.

## Supplementary Information

Below is the link to the electronic supplementary material.

Supplementary file1 (PDF 863 KB)

Supplementary file2 (XLSX 80 KB)
