# Supplementary material for: Metagenomic analysis of nepoviruses: diversity, evolution and identification of a genome region in members of subgroup A that appears to be important for host range
Source: Arch Virol. 2021 Aug 9;166(10):2789–801. doi: 10.1007/s00705-021-05111-0 (PMC8421298; doi:10.1007/s00705-021-05111-0)
Supplement: Supplementary file 1 — Supplementary file1 (PDF 863 KB) [file 705_2021_5111_MOESM1_ESM.pdf]

Metagenomic analysis of nepoviruses: diversity, evolution and identification of a genome region in members of subgroup A that appears to be important for host range

Hily JM[1, 2], Poulicard N[3], Kubina J[2], Reynard JS[4], Spilmont AS[1], Fuchs M[5], Lemaire O[2] and Vigne E[2]*.
1 IFV, Le Grau-Du-Roi, France
2 Université de Strasbourg, INRAE, SVQV UMR-A 1131, F-68000 Colmar, France
3 PHIM, Université Montpellier, IRD, INRAE, Cirad, SupAgro, Montpellier, France
4 Institute for Plant Production Science, Agroscope, 1260 Nyon, Switzerland
5 Cornell University, Geneva, NY, USA
* Corresponding author: emmanuelle.vigne@inrae.fr
Archives of Virology

Figure S1. Unrooted maximum likelihood trees of the complete set of sequences of the genus *Nepovirus* with 110 ORF1 and 167 ORF2 nucleotide sequences. Sequences from the genera *Fabavirus* (ORF1:BBW1_NC_005289 and BBW2_KJ789136; ORF2: BBW1_NC_005290 and BBW2_KJ789137) and *Comovirus* (ORF1: CPMV_NC_003549, ORF2: CPMV_NC_003550) were used as outgroups. Colors correspond to nepovirus species affiliation with red for subgroup A sequences, blue for subgroup sequences and green for subgroup C sequences. The scale bars show genetic distance.

Figure S2. Schematic representation of intra- (triangles) and inter-species (circles) recombination events along ORF1 (left panel) and ORF2 (right panel) nucleotide sequences of representative sequences of each of the three nepovirus subgroups. Maps of the two ORFs are not drawn to scale. Numbers within triangles and circles correspond to recombination events predicted at hot spot locations. Details and precise locations of recombination events are provided in Tables S5 and S6.

Figure S3. Maximum likelihood trees using all available (as of January 2020) nucleotide sequences corresponding to the coding regions 2A, MP and CP of arabis mosaic virus (ArMV) isolates. The list of ArMV sequences is provided in Table S8. Numbers at each node indicate bootstrap values based on 100 replicates. Scale bar show genetic distance. ArMV isolates infecting monocotyledonous plants were shown in red and those infecting dicotyledonous plants are in green.

Figure S4. Genetic diversity analyses of grapevine fanleaf virus (GFLV) isolates from different countries using a corpus of 40 ORF1 (left panel) and 80 ORF2 (right panel) nucleotide sequences. Colors correspond to different regions of the world with New World isolates in yellow, Old World isolates in green, French isolates in blue and isolates from the rest of the world in red. Graphics represent π (substitution/sites) and Tajima's D (D_T) for evolution along the ORF1 and ORF2 sequences. Colored bars with # and * correspond to genetic regions being statistically valid (P-values at 0.05 and 0.001), respectively.

Figure S5. Phylogenetic and diversity analyses of the CP coding region using a corpus of 102 nucleotide sequences of grapevine fanleaf virus (GFLV), arabis mosaic virus (ArMV) and grapevine deformation virus (GDefV) isolates with π (substitution/sites) shown along the sequence (a), phylogenetic relationships using maximum likelihood tree (b), and consensus amino acids obtained by aligning regions previously predicted and/or validate in nematode-mediated transmission (R1-R5) (c). GFLV isolates are in blue, all ArMV isolates in yellow grapevine-infecting ArMV isolates (*Vitis*-ArMV) in yellow, and non-grapevine-infecting ArMV isolates (Non-*Vitis*-ArMV) in red. For panel a, the tree CP domains (A, B, C) are delineated. For the phylogenetic tree, numbers at each node indicate bootstrap values based on 100 replicates and the scale bar shows genetic distance.

Figure S6. Comparative analysis of the six possible reading frames of ORF1 from 40 grapevine fanleaf virus (GFLV) and 17 arabis mosaic virus (ArMV) sequences. Graphic representation of π (substitution/sites) along the sequence (top) and of the +1, +2, +3, -1, -2, -3 reading frames with stop codons in blue and gaps in gray (bottom). Within each reading frame that consists of 57 lines or sequences, the first 40 lines correspond to GFLV sequences, followed by nine *Vitis*-infecting ArMV sequences and finally by eight non-*Vitis*-infecting ArMV sequences. GFLV sequences are in blue, *Vitis*-infecting ArMV sequences in green and non-*Vitis*-infecting ArMV in red.

Figure S7. Phylogenetic tree reconstructed from the alignment of the distal 148 and 149 amino acids of the RNA-dependent RNA polymerase (Pol) from grapevine fanleaf virus (GFLV) and arabis mosaic virus (ArMV) isolates. The scale bar below the tree shows genetic distance. GFLV sequences are delineated in a blue area, *Vitis*-infecting ArMV sequences in a green area and non-*Vitis*-infecting ArMV in a red area.

Figure S8. Alignment of consensus amino acid sequences of the C-terminal part of the polymerase (Pol) from grapevine fanleaf virus (GFLV) isolates, *Vitis*-infected isolates of arabis mosaic virus (ArMV) and non-*Vitis*-infecting ArMV isolates. Arrows indicate identical amino acids between grapevine viruses but different from ArMV isolates infecting plants other than grapevines. The blue rectangle shows the distal 148/149 aa in the Pol domain.

Figure S1

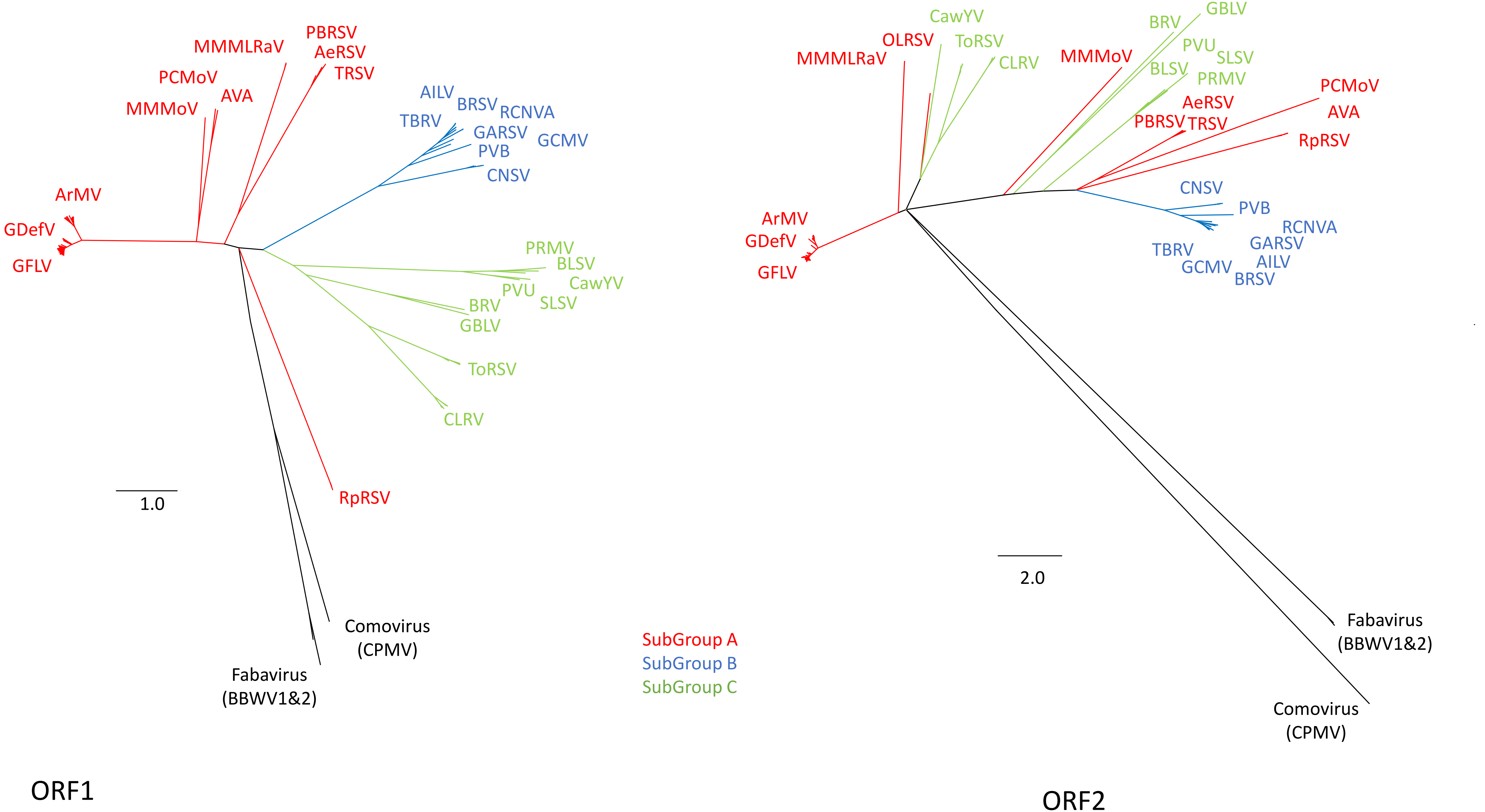

MMMLRaV
PBRSV
AeRSV
TRSV
PCMoV
AVA
MMMoV
ArMV
GDefV
GFLV
AILV
BRSV
RCNVA
TBRV
GARSV
GCMV
PVB
CNSV
PRMV
BLSV
CawYV
PVU
SLSV
BRV
GBLV
ToRSV
CLRV
RpRSV
1.0
Comovirus
(CPMV)
Fabavirus
(BBWV1&2)
ORF1
CawYV
OLRSV
ToRSV
MMMLRaV
CLRV
MMMoV
BRV
GBLV
PVU
SLSV
BLSV
PRMV
PCMoV
AeRSV
AVA
PBRSV
TRSV
RpRSV
CNSV
PVB
ArMV
GDefV
GFLV
RCNVA
GARSV
TBRV
AILV
GCMV
BRSV
2.0
Fabavirus
(BBWV1&2)
Comovirus
(CPMV)
ORF2
SubGroup A
SubGroup B
SubGroup C

Figure S2

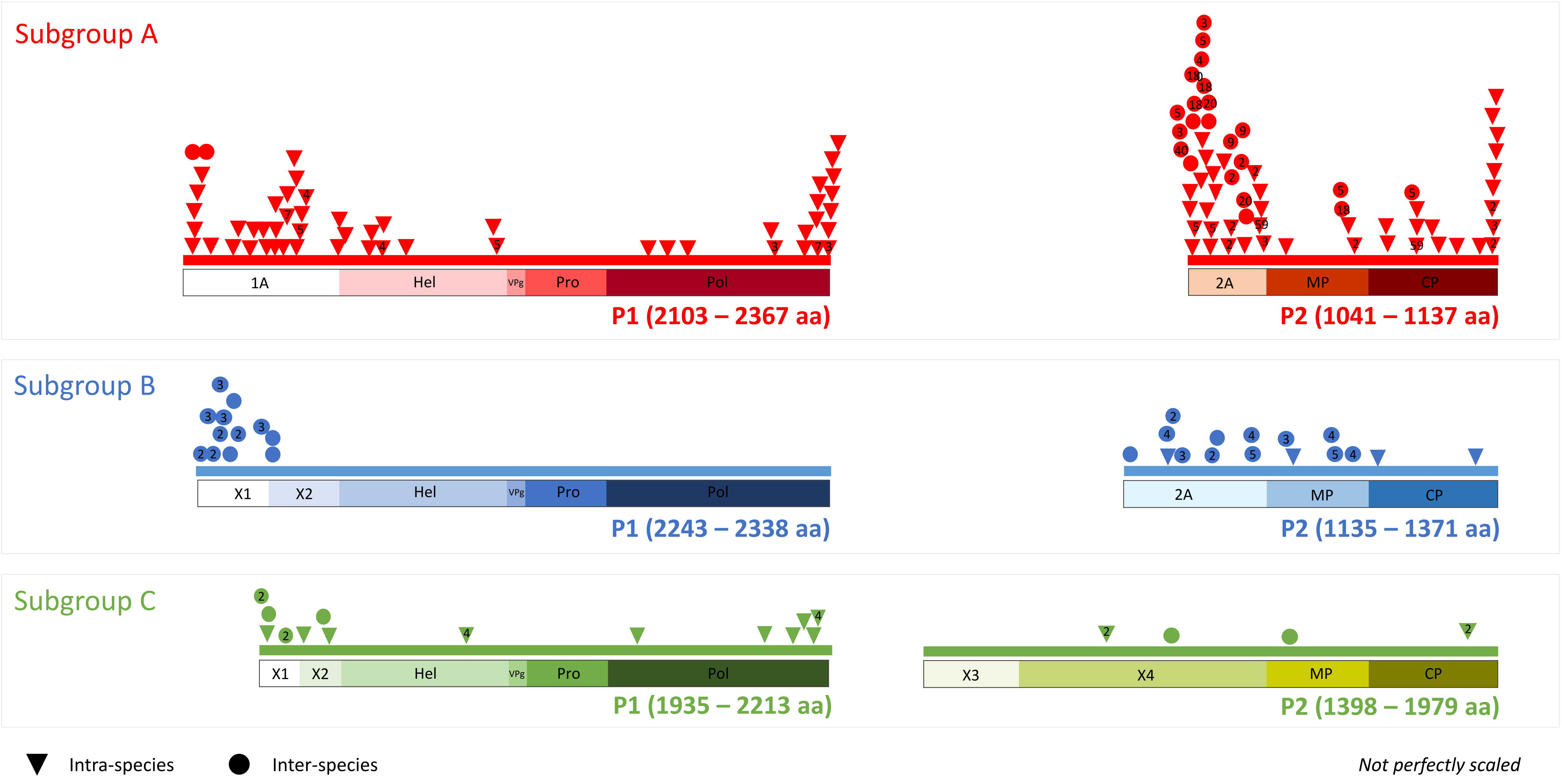
Subgroup A
1A
Hel
VPg
Pro
Pol
P1 (2103 – 2367 aa)
2A
MP
CP
P2 (1041 – 1137 aa)
Subgroup B
X1
X2
Hel
VPg
Pro
Pol
P1 (2243 – 2338 aa)
2A
MP
CP
P2 (1135 – 1371 aa)
Subgroup C
X1
X2
Hel
VPg
Pro
Pol
P1 (1935 – 2213 aa)
X3
X4
MP
CP
P2 (1398 – 1979 aa)
Intra-species
Inter-species
Not perfectly scaled

Figure S3

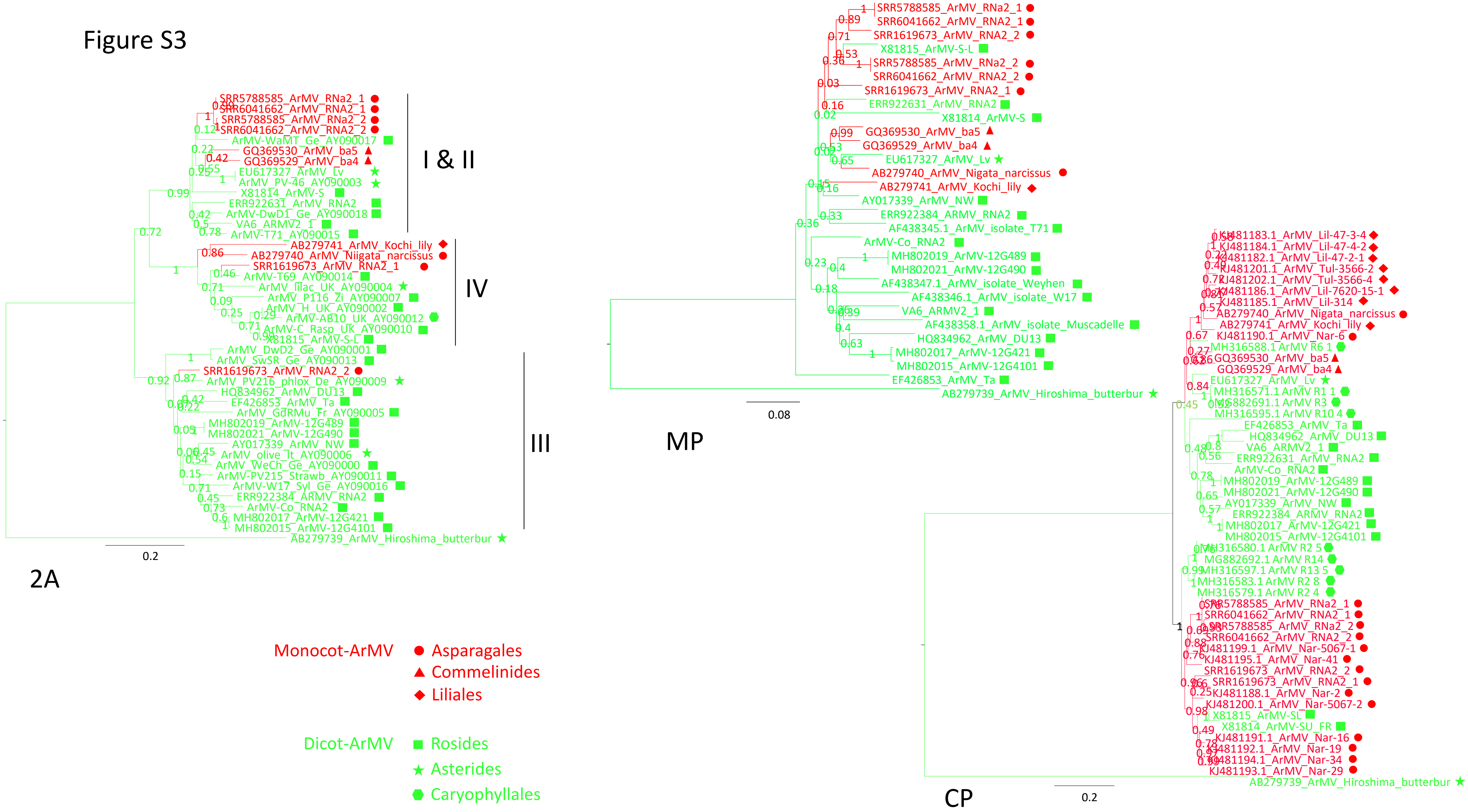
SRR5788585_ArMV_RNa2_1
SRR6041662_ArMV_RNA2_1
SRR5788585_ArMV_RNa2_2
SRR6041662_ArMV_RNA2_2
ArMV-WaMT_Ge_AY090017
GQ369530_ArMV_ba5
GQ369529_ArMV_ba4
EU617327_ArMV_Lv
ArMV_PV-46_AY090003
X81814_ArMV-S
ERR922631_ArMV_RNA2
ArMV-DwD1_Ge_AY090018
VA6_ARMV2_1
ArMV-T71_AY090015
I & II
AB279741_ArMV_Kochi_lily
AB279740_ArMV_Niigata_narcissus
SRR1619673_ArMV_RNA2_1
ArMV-T69_AY090014
ArMV_lilac_UK_AY090004
ArMV_P116_Zi_AY090007
ArMV_H_UK_AY090002
ArMV-AB10_UK_AY090012
ArMV-C_Rasp_UK_AY090010
X81815_ArMV-S-L
IV
ArMV_DwD2_Ge_AY090001
ArMV_SwSR_Ge_AY090013
SRR1619673_ArMV_RNA2_2
ArMV_PV216_phlox_De_AY090009
HQ834962_ArMV_DU13
EF426853_ArMV_Ta
ArMV_GdRMu_Fr_AY090005
MH802019_ArMV-12G489
MH802021_ArMV-12G490
AY017339_ArMV_NW
ArMV_olive_It_AY090006
ArMV_WeCh_Ge_AY090000
ArMV-PV215_Strawb_AY090011
ArMV-W17_Syl_Ge_AY090016
ERR922384_ARMV_RNA2
ArMV-Co_RNA2
MH802017_ArMV-12G421
MH802015_ArMV-12G4101
III
AB279739_ArMV_Hiroshima_butterbur
0.2
2A
SRR5788585_ArMV_RNa2_1
SRR6041662_ArMV_RNA2_1
SRR1619673_ArMV_RNA2_2
X81815_ArMV-S-L
SRR5788585_ArMV_RNa2_2
SRR6041662_ArMV_RNA2_2
SRR1619673_ArMV_RNA2_1
ERR922631_ArMV_RNA2
X81814_ArMV-S
GQ369530_ArMV_ba5
GQ369529_ArMV_ba4
EU617327_ArMV_Lv
AB279740_ArMV_Nigata_narcissus
AB279741_ArMV_Kochi_lily
AY017339_ArMV_NW
ERR922384_ARMV_RNA2
AF438345.1_ArMV_isolate_T71
ArMV-Co_RNA2
MH802019_ArMV-12G489
MH802021_ArMV-12G490
AF438347.1_ArMV_isolate_Weyhen
AF438346.1_ArMV_isolate_W17
VA6_ARMV2_1
AF438358.1_ArMV_isolate_Muscadelle
HQ834962_ArMV_DU13
MH802017_ArMV-12G421
MH802015_ArMV-12G4101
EF426853_ArMV_Ta
AB279739_ArMV_Hiroshima_butterbur
0.08
MP
KJ481183.1_ArMV_Lil-47-3-4
KJ481184.1_ArMV_Lil-47-4-2
KJ481182.1_ArMV_Lil-47-2-1
KJ481201.1_ArMV_Tul-3566-2
KJ481202.1_ArMV_Tul-3566-4
KJ481186.1_ArMV_Lil-7620-15-1
KJ481185.1_ArMV_Lil-314
AB279740_ArMV_Nigata_narcissus
AB279741_ArMV_Kochi_lily
KJ481190.1_ArMV_Nar-6
MH316588.1 ArMV R6 1
GQ369530_ArMV_ba5
GQ369529_ArMV_ba4
EU617327_ArMV_Lv
MH316571.1 ArMV R1 1
MG882691.1 ArMV R3
MH316595.1 ArMV R10 4
EF426853_ArMV_Ta
HQ834962_ArMV_DU13
VA6_ARMV2_1
ERR922631_ArMV_RNA2
ArMV-Co_RNA2
MH802019_ArMV-12G489
MH802021_ArMV-12G490
AY017339_ArMV_NW
ERR922384_ARMV_RNA2
MH802017_ArMV-12G421
MH802015_ArMV-12G4101
MH316580.1 ArMV R2 5
MG882692.1 ArMV R14
MH316597.1 ArMV R13 5
MH316583.1 ArMV R2 8
MH316579.1 ArMV R2 4
SRR5788585_ArMV_RNa2_1
SRR6041662_ArMV_RNA2_1
SRR5788585_ArMV_RNa2_2
SRR6041662_ArMV_RNA2_2
KJ481199.1_ArMV_Nar-5067-1
KJ481195.1_ArMV_Nar-41
SRR1619673_ArMV_RNA2_2
SRR1619673_ArMV_RNA2_1
KJ481188.1_ArMV_Nar-2
KJ481200.1_ArMV_Nar-5067-2
X81815_ArMV-SL
X81814_ArMV-SU_FR
KJ481191.1_ArMV_Nar-16
KJ481192.1_ArMV_Nar-19
KJ481194.1_ArMV_Nar-34
KJ481193.1_ArMV_Nar-29
AB279739_ArMV_Hiroshima_butterbur
0.2
CP
Monocot-ArMV
Asparagales
Commelinides
Liliales
Dicot-ArMV
Rosides
Asterides
Caryophyllales

Figure S4

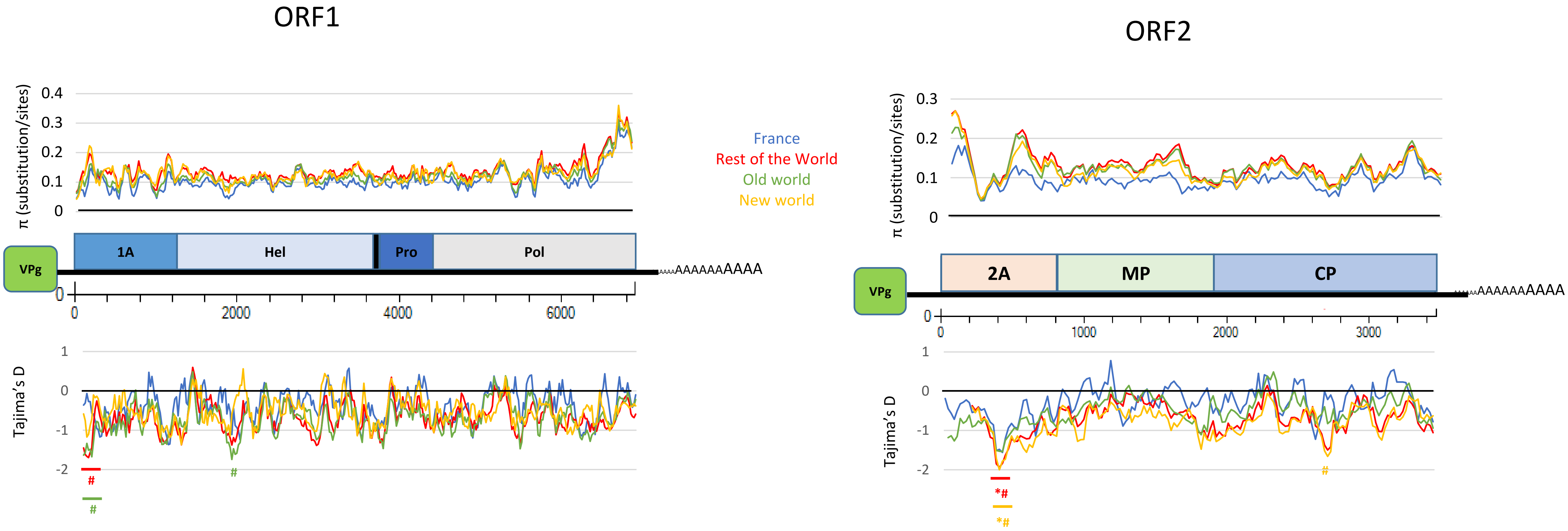
ORF1
ORF2
π (substitution/sites)
0.4
0.3
0.2
0.1
0
France
Rest of the World
Old world
New world
1A
Hel
Pro
Pol
VPg
AAAAAAAAAAAAAA
2000
4000
6000
Tajima's D
1
-1
-2
#
2A
MP
CP
1000
3000
*#

Figure S5

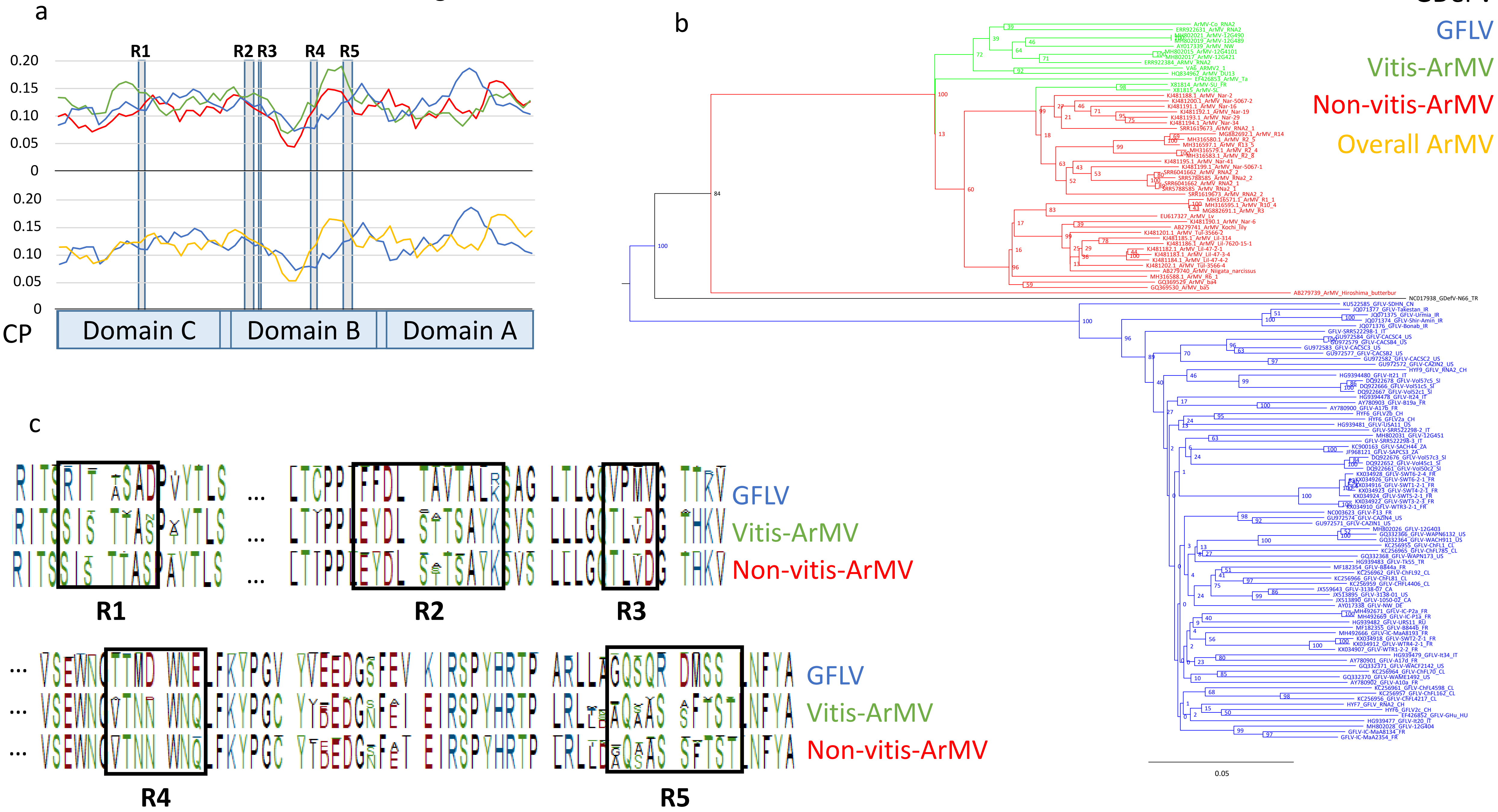

a
R1
R2
R3
R4
R5
0.20
0.15
0.10
0.05
0
CP
Domain C
Domain B
Domain A
b
GDeFV
GFLV
Vitis-ArMV
Non-vitis-ArMV
Overall ArMV
0.05
c
R1
R2
R3
R4
R5
GFLV
Vitis-ArMV
Non-vitis-ArMV

Figure S6

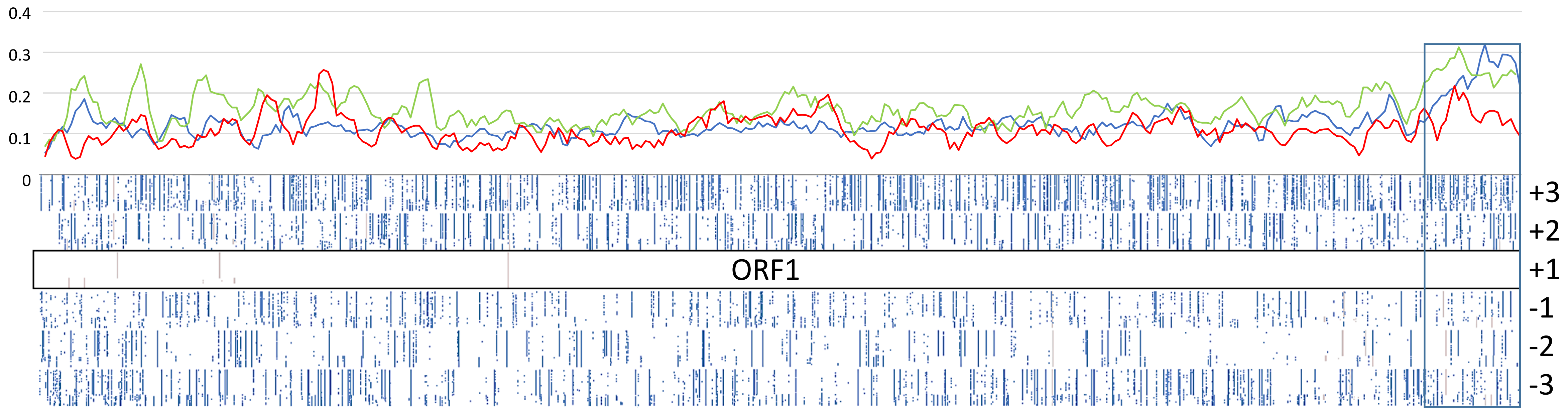

Figure S7

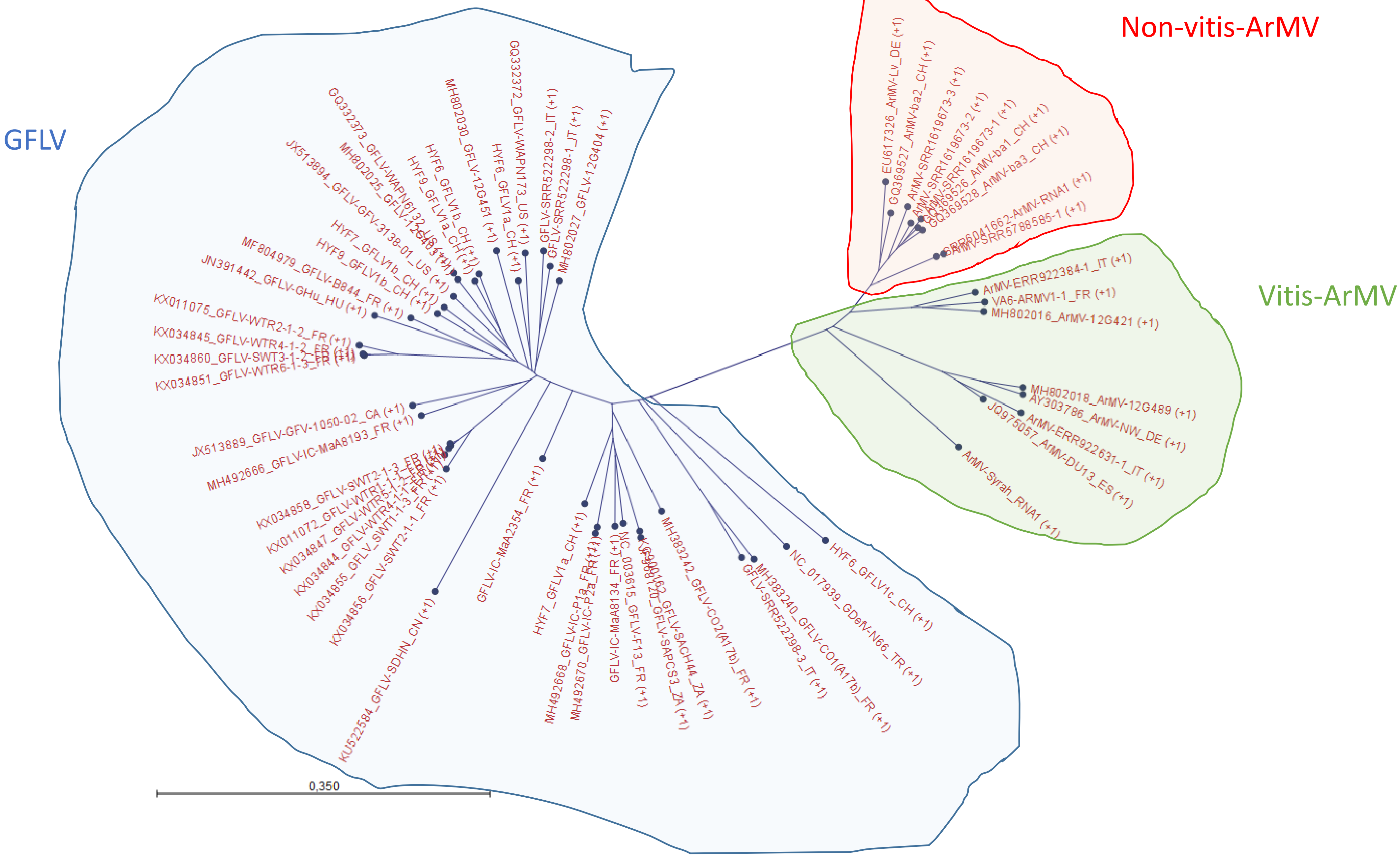

Last 148/149 aa of the Pol coding region

Figure S8

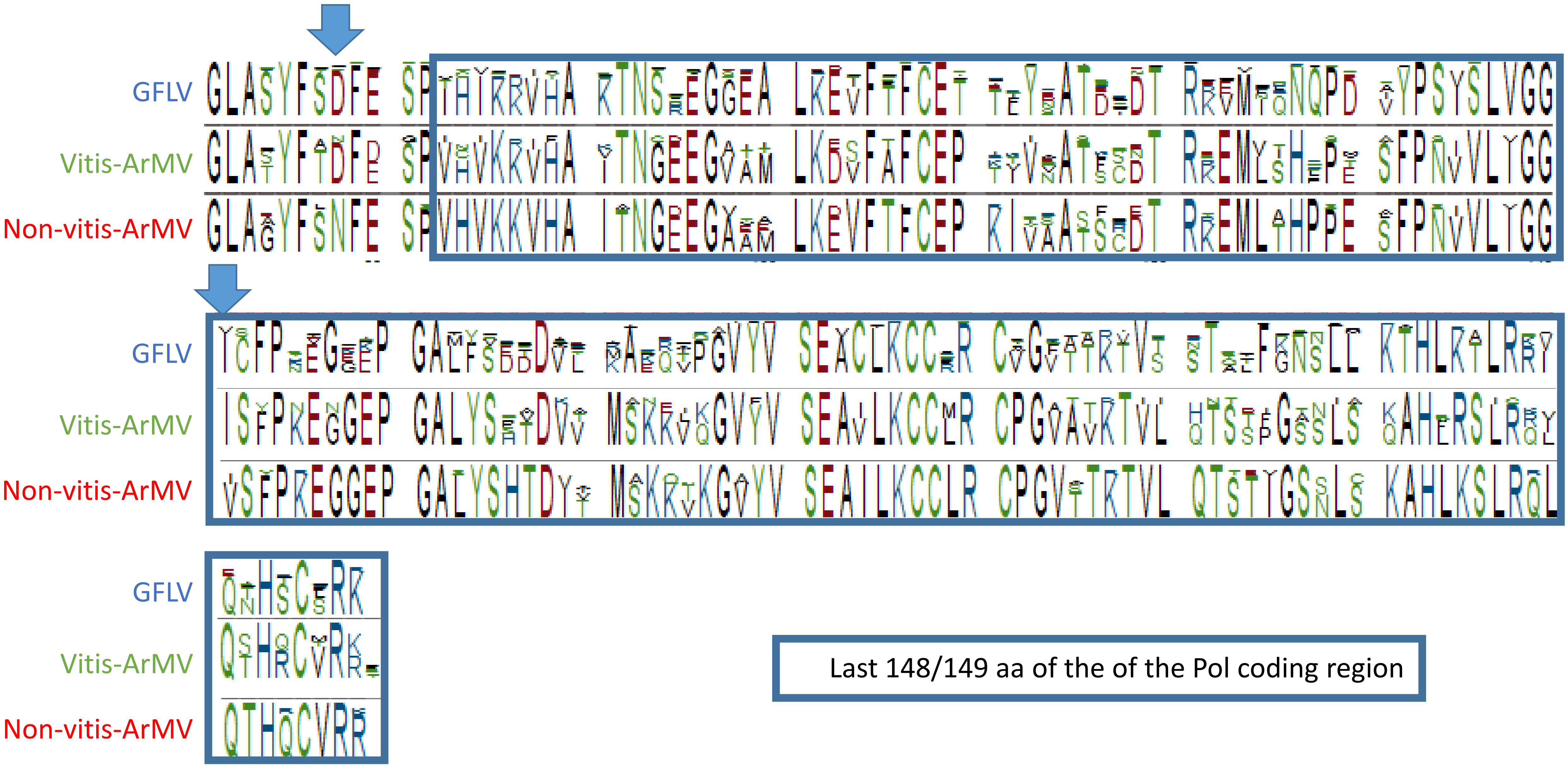

GFLV
Vitis-ArMV
Non-vitis-ArMV
GFLV
Vitis-ArMV
Non-vitis-ArMV
GFLV
Vitis-ArMV
Non-vitis-ArMV
Last 148/149 aa of the of the Pol coding region
